# Is recession bad for your mental health? The answer could be complex: evidence from the 2008 crisis in Spain

**DOI:** 10.1186/s12874-018-0538-2

**Published:** 2018-07-13

**Authors:** Joaquín Moncho, Pamela Pereyra-Zamora, Nayara Tamayo-Fonseca, Manuel Giron, Manuel Gómez-Beneyto, Andreu Nolasco

**Affiliations:** 1 Research Unit for the Analysis of Mortality and Health Statistics, University of Alicante, Campus de San Vicente del Raspeig s/n, Ap. 99, 03080 Alicante, Spain; 2 CIBERSAM, Instituto de Salud Carlos III, Madrid, Spain; 3 Teaching Unit of Psychiatry and Psychological Medicine, Department of Medicine, University of Valencia, Valencia, Spain

**Keywords:** Mental health, Population study, Economic recession, Unemployment, Prevalence

## Abstract

**Background:**

We explored the impact of 2008 recession on the prevalence of mental health problems in Spain.

**Methods:**

Repeated cross-sectional survey design. Datasets from 2006 and 2011 were used, and temporal change was examined. The study was conducted on the economically active population (16–64 years old). The two surveys included 29,478 and 21,007 people, obtaining a 96 and 89.6% response rate, respectively.

Multiple logistic regression models were adjusted to identify poor mental health risk factors. A standardisation analysis was performed to estimate the prevalence of people at risk of poor mental health (GHQ+).

**Results:**

The prevalence of GHQ+ following the crisis increased in men and decreased in women. Two logistic regression analyses identified GHQ+ risk factors. From 2006 to 2011, unemployment rose and income fell for both men and women, and there was a decline in the prevalence of somatic illness and limitations, factors associated with a higher prevalence of GHQ+. After controlling for age, the change in employment and income among men prompted an increase in the prevalence of GHQ+, while the change in somatic illness and limitations tended to mitigate this effect. After the recession, unemployed men showed a better level of somatic health. The same effects were not detected in women.

**Conclusions:**

The economic recession exerted a complex effect on mental health problems in men. The reduction of prevalence in women was not associated with changes in socioeconomic factors related to the economic crisis nor with changes in somatic health.

## Background

In his seminal work “Le suicide” (1897) [1], Durkheim argued that economic crises could increase psychiatric pathology. There are good grounds for thinking that this may be so, including consistent evidence that those who become unemployed during a recession may have worse mental health than those who do not [2]. Some studies have documented the direct effect of the economic recession on the general population’s mental health by comparing outcomes before and after the onset of the crisis [3–6]. However, the independent effect of changes in the distribution of socioeconomic and health risk factors has not been investigated. An economic recession does not exert a homogeneous effect on the population’s health status [7, 8], and the hypothesis of a “protective” effect of the downturn phase of the economic cycle on mental health has not been studied. This hypothesis is plausible because it is known that somatic morbidity is a risk factor for poor mental health [9] and that the population’s health status may improve during a recession, especially during periods of very rapid industrial contraction [10–12].

In 2008, following a period of economic growth based largely on the property market, Spain entered a severe and lasting economic crisis that generated a significant decline in macroeconomic activity (GDP) and a number of other factors with a clear impact on the population, such as an acute rise in unemployment and a deterioration in the standard of living. A substantial and rapid rise in unemployment can have negative effects on mental health, but positive effects on somatic health [10], giving rise to complex explanatory models. The Spanish National Health Surveys (SNHS) conducted in 2006 (pre-crisis) and 2011 (crisis) by the National Statistics Institute (Ministerio de Sanidad Servicios Sociales e Igualdad) consider a representative sample of the Spanish population. These surveys include Goldberg’s General Health Questionnaire (GHQ) and items relating to demographic, socioeconomic, support and health factors. The aim of this study was to analyse the impact of the economic recession on mental health, including possible pro-cyclical effects, and specifically, to investigate variation in the prevalence of mental health problems (GHQ+ caseness) in the general population in relation to variations in demographic and socioeconomic factors related to the crisis, as well as social support and health. The study was conducted in four phases: 1) estimation of the prevalence of GHQ+ and identification of the risk factors in 2006 and 2011; 2) estimation of changes in risk factor frequency and prevalence between the two periods; 3) analysis of the relationship between changes in risk factor frequency and changes in GHQ+ prevalence; 4) exploration of the relationship between unemployment and somatic health.

## Methods

### Data source

We used data from the Spanish National Health Survey [13] for two periods: 2006 (before the start of the crisis) and 2011 (during the crisis). National health surveys employ a multistage, stratified-random design to identify samples of adults. All residents in each household were screened and one person was selected at random for the interview. Data were collected between June 2006 and June 2007 (2006 survey), and between July 2011 and June 2012 (2011 survey). Face-to-face interviews were carried out at home by fully trained interviewers. We restricted the selected sample to the economically active population (16–64 years old). A total of 29,478 people aged > 15 years old responded to the SNSH health questionnaire in 2006, and 21,007 in 2011, obtaining a response rate of about 96 and 89.6%, respectively. Of these, 20,787 and 14,835, respectively, were aged between 16 and 64 years old, and these were the populations considered in the present study.

Due to the complex sample design, sample subjects were weighted to determine the number of subjects represented by each individual in the sample [14]. The weightings were included in the databases provided by the National Statistics Institute.

### Risk of poor mental health and explanatory variables

The risk of poor mental health was the outcome variable in 2006 and 2011 and was measured with the 12-item version of the General Health Questionnaire GHQ-12 [15, 16]. This screening measure detects probable psychiatric disorders. We used a 2-point scoring method, rating a problem as absent (0) or present (1) according to the method recommended by the developers of the questionnaire. Responses were summed, and subjects obtaining scores of 3 or above (out of 12) were classified as having poor mental health (GHQ+).

The same explanatory variables obtained in both 2006 and 2011 were:

- Sociodemographic: sex (male/female), age (16–34/35–49/50–64), marital status (single/married/separated or divorced/widowed), educational level (higher/other).
- Socioeconomic variables related to crises: occupation (employed/unemployed/other; such as: [retired, student, houseworker, disabled]) and household income (euros per month, high/average/low).
- Social support (measured by means of the 11-item Duke Social Support Index) [17]. Higher scores for individual items indicate better social support. Responses were summed, and subjects obtaining scores of ≤32 (out of 55) were classified as having low social support [18].
- Health variables: presence of a somatic illness confirmed by a doctor (yes/no), and presence of a limitation due exclusively to a somatic illness (yes/no). In order to determine the presence of somatic illness during the last year confirmed by a doctor, the surveys posed the following questions: “I’m going to read a list of illnesses or health problems; do you have or have you ever had any of them? If yes, was this in the last 12 months? Have you consulted a doctor about it?” The list was as follows: high blood pressure, heart attack, other cardiovascular diseases, varicose veins in the legs, osteoarthritis, arthritis or rheumatism, chronic back pain (cervical), chronic back pain (lumbar), chronic allergy, asthma, chronic bronchitis, diabetes, stomach or duodenal ulcer, urinary incontinence, high cholesterol, cataracts, chronic skin problems, chronic constipation, stroke, migraine or frequent headaches, hemorrhoids, cancer, osteoporosis, thyroid problems, prostate problems, menopausal problems. To analyze the change in prevalence we considered three groups: cardiovascular (high blood pressure, heart attack, other cardiovascular diseases, stroke), osteoarticular (osteoarthritis, arthritis or rheumatism, chronic back pain-cervical, chronic back pain-lumbar) and other. A fair agreement between questionnaire data and medical records has been well established in the case of common chronic disorders. [19, 20]. In order to determine the presence of a limitation due exclusively to a somatic illness, the surveys posed the following questions: “In the last six months, to what extent have you suffered limitations in activities of daily living?” Response options were: not limited at all/limited. For this question, only physical limitations were considered.

### Data analysis

Analyses were performed for men and for women. Multiple logistic regression models were fitted to allow calculation of adjusted odds ratios (ORs) and 95% confidence intervals (CIs).

A standardisation analysis was performed, considering age and variables that maintained a statistically significant relationship with a GHQ+ in the multivariate logistic regression analyses in the two surveys, and which showed a significant change in frequency between 2006 and 2011 for either sex. The expected prevalences of GHQ+ in 2011 were estimated as if the population had retained the same population structure observed in 2006. The difference between expected and observed prevalences in 2011 could indicate the effect of the crisis in terms of the controlled risk variables.

Specifically, to study possible changes in GHQ+ prevalence in men and women for the years 2006 and 2011 adjusted for age, employment status, income level, presence of a somatic illness and presence of limitations derived from a somatic illness, the population distribution in 2006 for each of the levels and sublevels of the previous variable categories was projected onto the 2011 population (2 sex categories × 3 age categories × 3 employment status categories × 3 income level categories × 2 somatic illness categories × 2 limitation categories = 216 cells or levels) as follows:

$$ {P}_{e_i}^{2011}=\frac{P_i^{2006}}{\sum {P}_i^{2006}}\left(\sum {P}_i^{2011}\right)\kern1em ;\kern1em i=1,2,3\dots ..,216 $$

Where:

${P}_{e_i}^{2011}$ = expected population in 2011 in level i.

${P}_i^{2006}$ = observed population in 2006 in level i.

${P}_i^{2011}$ = observed population in 2011 in level i.

Once the 2011 population had been estimated in each of the cells, the observed prevalence in 2011 was applied at the same level of disaggregation to obtain the expected GHQ+ cases and the corresponding prevalence, as described below:

$$ {n_{e\_ GHQ+}}_i^{2011}={P}_{e_i}^{2011}\cdot {prev}_i^{2011}={P}_{e_i}^{2011}\cdot \frac{{n_{GHQ+}}_i^{2011}}{P_i^{2011}}\kern1em ;\kern1em i=1,2,3,\dots, 216 $$

$$ {prev}_{e,i}^{2011}=\frac{{n_{e\_ GHQ+}}_i^{2011}}{P_{e_i}^{2011}} $$

Where:

${n_{e\_ GHQ+}}_i^{2011}$ = expected GHQ+ frequency in 2011 in level i.

${prev}_i^{2011}$ = observed GHQ+ prevalence in 2011 in level i.

${n_{GHQ+}}_i^{2011}$ = observed GHQ+ frequency in 2011 in level i.

Lastly, global prevalence was calculated disaggregated by sex, adding all previous cells, as follows:

$$ {prev}_{e, total}^{2011}=\frac{\sum {n_{e\_ GHQ+}}_i^{2011}}{\sum {P}_{e_i}^{2011}} $$

In addition, expected prevalences were calculated adjusting for sex, age, income level and employment status, and for sex, age, somatic illness and limitations.

This standardisation system made it possible to distinguish between: (1) changes in global prevalence due to variations in population structure between 2006 and 2011 (in terms of the variables considered), and (2) changes in prevalence that could not be explained by changes in the population structure. Furthermore, variation in variables for which a significant change in the population distribution was observed between the two periods under consideration could be related to variations in global GHQ+ prevalence.

## Results

### Relationship between the risk of poor mental health and sociodemographic, socioeconomic, support and comorbidity variables

Table 1 shows the estimations of GHQ+ prevalence together with the adjusted odds ratios using multivariate logistic regression, and the frequencies of the different risk factors in 2006 and 2011. The prevalence of GHQ+ increased in men but decreased in women. For both men and women, being a GHQ+ case presented a statistically significant association (*p* < 0.05) with a low educational level (2006 survey), being divorced/separated (2006), low social support (2006, 2011), unemployment (2006, 2011), low and average household income (2006, 2011), having a somatic illness (2006, 2011) and the presence of a limitation arising from a somatic illness (2006, 2011).

**Table 1:** Prevalence of risk of mental health problems (GHQ+), odds ratios (OR) and 95% confidence intervals (95% CI) for the association between GHQ+ and other explanatory variables, and distribution of population, by sex and year of survey

Men				
Prevalence of Total Mental Health Problems	2006 (*n* = 7681), 14.5% (13.8, 15.2)		2011 (*n* = 5479), 16.9% (16.0, 17.7)	
	OR (95% CI)		Distribution of population (%) (95%CI)	
	2006	2011	2006	2011
Age	*p* < 0.001	*p* < 0.001		
16–34	1	1	41.6 (40.6, 42.5)	36.6 (35.5, 37.7)
35–49	0.93 (0.79, 1.10)	1.42^c^ (1.17,1.73)	34.9 (34.0, 35.8)	36.6 (35.5, 37.7)
50–64	0.62^c^ (0.51,0.76)	0.82 (0.65, 1.03)	23.5 (22.7, 24.3)	26.8 (25.8, 27.8)
Educational level	*p* = 0.011	*p* = 0.154		
Higher/university	1	1	28.0 (27.2, 28.9)	24.5 (23.5, 25.5)
Others	1.21^a^ (1.04, 1.41)	1.16 (0.95,1.41)	72.0 (71.1, 72.8)	75.5 (74.5, 76.5)
Marital status	*p* < 0.001	*p* = 0.540		
Single	1	1	43.0 (42.0, 43.9)	41.0 (39.9, 42.1)
Married	1.05 (0.89, 1.23)	1.10 (0.91, 1.32)	52.7 (51.7, 53.8)	54.3 (53.2, 55.5)
Divorced/separated	2.07^c^ (1.54, 2.78)	1.10 (0.76, 1.59)	3.6 (3.3, 4.0)	4.1 (3.6, 4.5)
Widowed	0.78 (0.36, 1.69)	1.66 (0.75, 3.68)	0.7 (0.5, 0.9)	0.6 (0.5, 0.8)
Social support	*p* < 0.001	*p* = 0.004		
High	1	1	97.1 (96.8, 97.4)	97.4 (97.1, 97.8)
Low	5.05^c^(3.90, 6.55)	1.71^b^ (1.19, 2.46)	2.9 (2.6, 3.2)	2.6 (2.2, 2.9)
Employment status	*p* < 0.001	*p* < 0.001		
Employed	1	1	74.9 (74.1, 75.7)	61.3 (60.2, 62.4)
Unemployed	2.49^c^(2.35, 3.06)	2.52^c^ (2.09,3.05)	7.6 (7.1, 8.1)	20.7 (19.8, 21.6)
Other activity	1.56^c^ (1.34, 1.86)	1.85^c^ (1.50,2.28)	17.5 (16.8, 18.2)	18.0 (17.2, 18.9)
Household income	*p* = 0.007	*p* < 0.001		
High	1	1	36.6 (35.6, 37.5)	38.7 (37.6, 39.8)
Average	0.97 (0.84, 1.12)	1.33^b^ (1.10, 1.61)	50.1 (49.2, 51.1)	35.6 (34.5, 36.7)
Low	1.28^a^ (1.06, 1.56)	1.91^c^(1.55,2.36)	13.3 (12.7, 14.0)	25.7 (24.7, 26.6)
Somatic morbidity	*p* < 0.001	*p* < 0.001		
No	1	1	43.8 (42.8, 44.7)	47.9 (46.8, 49.1)
Yes	2.17^c^(1.88, 2.49)	1.79^c^ (1.52,2.10)	56.2 (55.3, 57.2)	52.1 (50.9, 53.2)
Limitation derived from somatic morbidity	*p* < 0.001	*p* < 0.001		
No	1	1	86.9 (86.3, 87.6)	89.5 (88.8, 90.2)
Yes	2.50^c^ (2.15, 2.91)	3.18^c^ (2.61,3.88)	13.1 (12.4, 13.7)	10.5 (9.8, 11.2)
Women				
Prevalence of Total Mental Health Problems	2006 (*n* = 11,035), 24.4% (23.6, 25.2)		2011 (*n* = 5522), 22.6% (21.7, 23.6)	
	OR (95% CI)		Distribution of population (%) (95%CI)	
	2006	2011	2006	2011
Age	*p* = 0.201	*p* = 0.069		
16–34	1	1	40.1 (39.2, 41.0)	35.6 (34.5, 36.7)
35–49	1.03 (0.90, 1.18)	1.24^a^ (1.03, 1.49)	34.8 (33.9, 35.7)	36.8 (35.8, 37.9)
50–64	0.91 (0.78, 1.07)	1.17 (0.95, 1.44)	25.1 (24.2, 25.9)	27.5 (26.5, 28.5)
Educational level	*p* < 0.001	*p* = 0.568		
Higher/university	1	1	26.9 (26.0, 27.7)	27.2 (26.1, 28.2)
Others	1.36^c^ (1.20, 1.55)	1.05 (0.89, 1.25)	73.1 (72.3, 74.0)	72.8 (71.8, 73.9)
Marital status	*p* < 0.001	*p* = 0.015		
Single	1	1	32.6 (31.7, 33.5)	34.9 (33.8, 36.0)
Married	0.92 (0.81, 1.05)	0.85 (0.71, 1.01)	59.3 (58.3, 60.2)	55.6 (54.4, 56.7)
Divorced/separated	1.30^a^ (1.03, 1.64)	1.13 (0.86, 1.48)	5.5 (5.0, 5.9)	6.7 (6.2, 7.3)
Widowed	1.45^a^(1.07, 1.98)	1.25 (0.86, 1.82)	2.7 (2.4, 3.0)	2.8 (2.4, 3.2)
Social support	*p* < 0.001	*p* < 0.001		
High	1	1	97.0 (96.7, 97.3)	96.6 (96.2, 97.0)
Low	4.31^c^(3.34, 5.56)	3.41 (2.51, 4.63)	3.0 (2.7, 3.3)	3.4 (3.0, 3.8)
Employment status	*p* < 0.001	*p* = 0.001		
Employed	1	1	52.3 (51.3, 53.3)	51.2 (50.0, 52.3)
Unemployed	1.38^c^ (1.17, 1.63)	1.44^c^(1.19,1.74)	10.1 (9.6, 10.7)	16.1 (15.3, 17.0)
Other activity	1.05 (0.94, 1.18)	1.12 (0.95, 1.32)	37.6 (36.6, 38.5)	32.7 (31.6, 33.7)
Household income	*p* < 0.001	*p* < 0.001		
High	1	1	32.2 (31.3, 33.1)	35.1 (34.0, 36.2)
Average	1.24^b^ (1.09, 1.40)	1.30^b^(1.10, 1.55)	52.1 (51.1, 53.0)	35.4 (34.4, 36.5)
Low	1.83^c^ (1.56, 2.15)	1.61^c^(1.33,1.94)	15.7 (15.0, 16.4)	29.5 (28.4, 30.5)
Somatic morbidity	*p* < 0.001	*p* < 0.001		
No	1	1	31.9 (31.0, 32.8)	37.8 (36.7, 38.9)
Yes	2.06^c^ (1.81, 2.33)	2.18^c^ (1.85,2.56)	68.1 (67.2, 69.0)	62.2 (61.1, 63.3)
Limitation derived from somatic morbidity	*p* < 0.001	*p* < 0.001		
No	1	1	84.7 (84.0, 85.4)	85.8 (85.0, 86.6)
Yes	2.07^c^ (1.82, 2.35)	2.53^c^ (2.14,3.00)	15.3 (14.6, 16.0)	14.2 (13.4, 15.0)

^a^*p* < 0.05, ^b^*p* < 0.01, ^c^*p* < 0.001

For the latter 4 risk factors (income, unemployment, somatic illness, limitation derived from somatic illness), differences in the distribution of frequencies were observed between 2006 and 2011 (Table 1). Frequencies of unemployed people and people living in low-income households increased, and frequencies of those living in average-income households and those with a somatic illness and a limitation deriving from a somatic illness decreased.

### Effect of changes in the frequency of socioeconomic and somatic health variables on the change in GHQ+ prevalence

The variables included in the standardisation analysis were employment status, household income, presence of a somatic illness and presence of a limitation. A slight increase was observed in 2011 in the percentage of people who were divorced/separated; however, the marital status variable was not included in the analysis due to the small number of subjects. Table 2 shows the GHQ+ prevalences observed for the different levels of explanatory variables. Once adjusted for age, a higher GHQ+ prevalence was observed among both men and women in 2006 and 2011 in the higher risk levels (unemployment, low income level, somatic illness, presence of limitation).

**Table 2:** Percentage of population (Pop %) and prevalences observed in 2006 and 2011 (P_o_) and expected in 2011 (P_e_) according to different standardisation models by sex

	2006		2011		2011		
	Pop %	P_o_	Pop %	P_o_	Model 1^a^ P_e_	Model 2^a^ P_e_	Model 3^a^ P_e_
Men							
MHP (95% CI)		14.5 (13.8;15.2)		16.9 (16.0, 17.7)	14.8 (14.0, 15.6)	17.8 (16.9, 8.7)	15.6 (14.8, 16.4)
Age							
16–34	41.6	13.6	36.6	14.2	11.9	14.9	12.2
35–49	34.9	15.0	36.6	19.4	16.4	20.8	17.7
50–64	23.5	15.3	26.8	17.0	17.7	18.6	18.7
Household Income							
low	11.9	23.1	18.9	28.5	24.6		26.7
average	44.7	13.6	26.3	17.1	15.6		16.1
high	32.5	12.2	28.6	11.6	10.9		11.2
no information	10.9	15.7	26.2	14.1	12.5		14.6
Occupation							
Employed	74.9	12.1	61.3	12.0	12.3		12.9
Unemployed	7.6	28.1	20.7	28.4	25.9		28.0
Others	17.5	18.6	18.0	20.2	20.6		21.9
Somatic Illness							
yes	56.1	19.0	52.1	21.6		22.5	19.4
no	43.9	8.7	47.9	11.7		11.8	10.8
Limitation							
yes	13.1	29.7	10.5	41.2		37.9	37.8
no	86.9	12.2	89.5	13.5		14.8	12.3
Women							
MHP (95% CI)		24.4 (23.6, 25.2)		22.6 (21.7, 23.6)	22.3 (21.3, 23.2)	23.4 (22.4, 24.3)	23.3 (22.4, 24.3)
Age							
16–34	40.1	21.4	35.6	17.5	17.9	18.9	19.3
35–49	34.8	24.8	36.8	24.0	22.9	24.7	23.8
50–64	25.0	28.6	27.5	27.5	28.4	28.7	29.2
Household Income							
low	13.6	36.4	21.1	30.9	30.8		31.9
average	45.4	24.8	25.4	23.6	23.3		24.8
high	28.0	18.6	25.2	17.7	17.6		17.9
no information	13.0	22.7	28.3	19.9	19.7		20.9
Occupation							
Employed	52.3	22.1	51.2	18.8	19.0		20.1
Unemployed	10.1	30.1	16.1	30.5	27.4		29.8
Others	37.5	26.1	32.7	24.8	25.3		26.1
Somatic Illness							
yes	68.1	29.0	62.2	28.6		28.2	28.4
no	31.9	14.5	37.8	12.9		13.0	12.3
Limitation							
yes	15.3	40.1	14.2	48.5		42.8	43.9
no	84.7	21.5	85.8	17.3		19.9	19.6

(^a^) Model 1 = standardised prevalence by age, household income and occupation

Model 2 = standardised prevalence by age, somatic illness and limitation

Model 3 = standardised prevalence by age, household income, occupation, somatic illness and limitation

Since the percentage distribution of risk factors varied differently between the 2 periods (the percentage of unemployed people and low-income households increased while the percentage of people with somatic illness and limitation derived from somatic illness decreased), its impact on the expected prevalence of GHQ+ in 2011 also had different effects. In 2011, the standardised (expected) GHQ+ prevalence in men by age and socioeconomic risk variables related to the crisis (employment status and income) was 14.8%, lower than the prevalence actually observed in 2011 (16.9%) (Table 2, Model 1). This indicates that the rise in unemployment and low-income households in both periods may explain the increase in observed GHQ+ prevalence. However, the standardised prevalence by age and variables related to somatic illness was 17.8%, higher than the observed prevalence (Table 2, Model 2). This indicates that the decrease in the frequency of having a somatic illness and limitations between the two periods may explain a decrease in observed GHQ+ prevalence. Model 3 (Table 2) corresponds to the prevalence of GHQ+ in 2011 standardised for all the above variables (age, employment status, income, somatic illness, limitation), which was 15.6%, lower than the observed prevalence in 2011 (16.9%). For women, the models adjusted for age and these two groups of risk factors did not show any major differences between expected and observed prevalence.

### Employment status and somatic illness

Table 3 shows a statistically significant reduction of somatic diseases in unemployed men and employed women.

**Table 3:** Prevalences of somatic illness and adjusted OR (95% IC) for the association between ocupational activity and somatic illness in economically active population

	Employed			Unemployed		
	2006	2011	diference	2006	2011	diference
Men						
Cardiovascular	14.6 (13.8, 15.3)	15.8 (14.7, 16.8)	1.2	14.1 (11.7, 16.5)	15.8 (13.9, 17.6)	1.7
Osteoarticular	23.1 (22.2, 24.0)	19.2 (18.0, 20.3)	−3,9^b^	27.9 (24.8, 31.0)	17.4 (15.8, 18.3)	−10,5^b^
Other	42.1 (41.0, 43.2)	41.0 (39.6, 42.4)	−1,1	42.1 (38.7, 45.5)	37.4 (35.0, 39.8)	−4,7
Total	55.2 (54.1, 56.3)	52.9 (51.5, 54.4)	−2.3	57.8 (54.3, 61.2)	48.6 (46.1, 51.1)	−9.2^b^
Adjusted Odds ratio ^a^	1	1		0.97 (0.81, 1.16)	0.85 (0.72, 0.99)	
Women						
Cardiovascular	12.3 (11.4, 13.2)	10.2 (9.3, 11.2)	−2,1^b^	12.8 (10.8, 14.9)	14.4 (12.4, 16.4)	1,6
Osteoarticular	34.4 (33.1, 35.6)	29.1 (27.6, 30.5)	−5,3^b^	38.4 (35.5, 41.4)	30.1 (27.5, 32.6)	−8,3^b^
Other	55,7 (54.3, 57.0)	51,0 (49.4, 52.5)	−4,7^b^	56.5 (53.5, 59.5)	52.3 (49.5, 55.2)	−4,2
Total	66.0 (64.7, 67.2)	60.5 (58.9, 62.0)	−5.5^b^	67.6 (64.8, 70.4)	62.6 (59.8, 65.3)	−5.0
Adjusted Odds ratio ^a^	1	1		1.03 (0.87, 1.20)	1.09 (0.91, 1.29)	

^a^Multivariate Logistic regression analysis controlling for age, social support, educational level, marital status, GHQ caseness, and household income

^b^Statistically significant change between 2006 and 2011

Using the presence of somatic illness as the response variable, the multivariate logistic regression models built for the periods 2006 and 2011 showed no statistically significant association between employment status and somatic health before the economic crisis in both sexes. However, during the crisis, unemployed men were significantly less likely to report poor health compared with employed people. There was a positive and statistically significant relationship between been a member of any of the three groups of somatic illness and being classified as GHQ+ case, in both 2006 and 2011. The prevalence of osteoarticular illnesses diminished in both sexes, the reduction being higher in the unemployed group. The prevalence of cardiovascular and other diseases diminished significantly in employed women.

## Discussion

This study has revealed that the 2008 economic recession has exerted a complex effect on the prevalence of men at risk of poor mental health. Compared with the pre-crisis period, the period of recession was characterised by an increase in unemployment and financial difficulties and by a decrease in health problems, factors all of them related with a higher prevalence of GHQ+ before and after the crisis. The increase in socioeconomic factors related to the crisis had a significant impact on the increased prevalence of GHQ+ cases. In contrast, the decrease in somatic illness and somatic illness derived limitations during the recession tended to mitigate this prevalence. Overall, these opposite effects tended to neutralise the crisis effect on the prevalence of GHQ+ cases in both men and women, indicating that the absolute increase in the observed prevalence of GHQ+ cases in men and its reduction in women must be due to factors other than those studied here. Second, although the prevalence of somatic illnesses decreased in all employment status categories after the onset of the crisis, this reduction was more marked among the unemployed men.

The effect of the economic recession on the risk of poor mental health in economically active men, exerted through a rise in unemployment and a drop in household income, is consistent with the model that assumes that the negative impact on health of a contracting economy is the result of undesirable consequences associated with job loss (anticipated or actual job loss, income reduction, difficulty in paying bills) [21–23]. Our results do not agree with those obtained in studies conducted in England [3, 4], where no relationship was found between a change in employment status and an increase in poor mental health in men. This discrepancy may be due to differences between the increase in unemployment in England and Spain (much lower in the former), the periods studied, and definitions of poor mental health and analysis methods.

This study provides evidence of the “protective” effect of economic recession on the risk of poor mental health among economically active men through a reduction in somatic health problems. This decrease in health problems during an economic recession has been reported in other studies conducting comparing outcomes before and after the crisis [11, 12].

This study has shown that unemployment exerts a direct effect increasing the prevalence of GHQ+ in men. However, this effect may have been mitigated by a decrease in somatic illness among the newly unemployed. The difference between the adjusted OR in 2006 and 2011 may be explained by the intake of previously healthy people among the newly unemployed, a phenomenon to be expected in periods of very rapid industrial contraction [10]; by a reduction in negative, unexpected health consequences of economic processes such as an increase in work, promotion at work and income level as anomic processes [22], or by a reduction in somatic problems due to the avoidance of jobs that endanger health (for example, due to a reduction in employment and workplace accidents in construction) [24, 25]. According to the Spanish Labor Ministry, total workplace accidents in Spain decreased between 2006 and 2011 by 68.5% in the construction sector and 34.4% in the other sectors, a decrease that may be related to the housing recession and the decline in global economic activity. This is consistent with the observed decrease in the prevalence of somatic limitations and osteoarticular ilness. On the other hand, it is known that changes in time and the limitation of income during economic expansions and contractions affect people’s behaviours, so that as the working time decreases during a recession; recreational exercise, hours of sleep, time devoted to the care of children and housework increases. However, it seems that these activities would not compensate for the decrease in work-related exertion due to job-loss, resulting in a cumulative decrease in net physical exertion. These effects would be of greater magnitude in men with a lower level of education, which is consistent with the fact that the sectors most affected by unemployment are the manufacturing industry and construction [26].

In women, the lack of effect of economic recession on risk of poor mental health adjusting for socioeconomic and health factors is a finding consistent with previous studies [3, 23]. Since women have access to alternative roles that to some extent may serve as a substitute for employment, unemployment may entail less social stigma (and self-reproach) for women than for men [27]. In addition, there may be other factors at play that reduce the risk of poor mental health in women, offsetting the potential impact of unemployment and loss of income. These might include a reduction in the relative distance between the values of women’s social role as a source of unpaid care with respect to the value of men’s role as breadwinner. Thus, the mental health consequences of economic crises are context dependent.

This study has several strengths. Our state level probability sample supports the generalisability of this study. We used comparable surveys, and took advantage of the “natural experiment” caused by the economic recession. However, some limitations should be noted. First, because of the cross-sectional rather than longitudinal design, it could be argued that prior poor mental health leads to loss of employment and income; however, the intensity of the economic recession and its known and rapid impact on loss of employment and income renders it unlikely that the relationship between socioeconomic factors related to the crisis and poor mental health is the reverse of that hypothesised. Nor can it be ruled out that some unmeasured factor, such as a previous individual psychological vulnerability, could be the source of both the loss of employment or household income and worsening poor mental health. However, this hypothesis is unlikely. It is doubtful that such a vulnerability factor would vary significantly over time, and so their prevalence in the studied population samples. On the other hand, as it has been indicated in various studies [28–30], social support, resilience and coping strategies would have a mitigating effect on the negative consequences of unemployment on individuals’ mental health and could have contributed to the reduction of the prevalence value of GHQ+ in 2011. Response bias (unrelated to sex, age or socioeconomic factors) could explain the relationship between unemployment and self-reports of somatic illness or limitation. Second, our study does not provide information on job insecurity, work overload or pay cuts, factors that may have been favored by the crisis nor about the type of occupation whose relationship with mental health is known.

Given that the health effects of economic recessions tend to be lagged [31], that long-term studies have documented the cumulative negative physical and psychological effects of sustained economic hardship [32], and the continuing effect of the crisis on unemployment and poverty, the effects observed in this study may intensify and change over time. It has been suggested that the long-term effects of an economic recession may increase the demand for care [8]. However, since people experiencing economic difficulties are more likely to have poorer mental health and might be less likely to receive health care, programmes may be necessary to mitigate the impact of the crisis at the population level or to provide mental health services to groups at risk. Finally, the recovery of economic activity in a new context of job insecurity and loss of rights does not guarantee an improvement in the population’s mental health.

## Conclusions

This study has found evidences that the economic recession exerted a complex effect on mental health problems in men. The reduction of prevalence in women was not associated with changes in socioeconomic factors related to the economic crisis nor with changes in somatic health.

## Abbreviations

95% CI: 95% confidence interval
GHQ-12: 12-item General Health Questionnaire
OR: Odds Ratios
SNHS: National Health Surveys
